# Association of problems, coping styles, and preferred online activity with depression, anxiety, and other psychological disorders in Turkish adolescents diagnosed with chronic kidney disease

**DOI:** 10.1007/s00467-024-06391-9

**Published:** 2024-05-21

**Authors:** Ibrahim Kandemir, Kemal Gudek, Aylin Yetim Sahin, Melike Tugrul Aksakal, Elif Kucuk, Zeynep Nagehan Yuruk Yildirim, Alev Yilmaz, Ahmet Nayir, Firdevs Bas

**Affiliations:** 1 Department of Pediatrics, Faculty of Medicine, Biruni University, Istanbul, Turkey; 2 Medical Social Service Unit, Faculty of Medicine, Istanbul University, Istanbul, Turkey; 3 Division of Adolescent Medicine, Department of Pediatrics, Faculty of Medicine, Istanbul University, Istanbul, Turkey; 4 Department of Psychiatry, Yuksek Ihtisas University, Ankara, Turkey; 5 Division of Pediatric Nephrology, Department of Pediatrics, Faculty of Medicine, Istanbul University, Istanbul, Turkey

**Keywords:** Chronic kidney disease, Depression, Anxiety, Adolescent

## Abstract

**Background:**

To assess depression, anxiety, and other psychological disorders in adolescents with chronic kidney disease (CKD) and determine the significant factors and the effect of digital media use on its scores among these patient groups.

**Methods:**

The study was conducted as a cross-sectional study and included 84 adolescents with CKD and 68 healthy controls. The participants completed the Revised Child Anxiety and Depression Scale (RCADS). We recorded their age, gender, the most problematic issue in their lives, coping methods with problems, and online applications they prefer in their leisure time.

**Results:**

Elevated rates (scores > 70) of separation anxiety, panic disorder, obsession, depression, total anxiety, and total depression scales were statistically higher in the CKD group. Separation anxiety, panic disorder, obsession, total anxiety, and total depression scales were higher in girls, and panic disorder, obsession, depression, total anxiety, and total depression scores were higher in younger ages in multivariate analysis. In the CKD group, family issues/problems increased panic disorder, obsession, depression, total anxiety, and total depression scales. Crying in tears/yelling response in children while facing a problem was associated with increased separation anxiety and social phobia rates. Also, preferring video applications was associated with separation anxiety and messaging applications with depression, total anxiety, and total depression.

**Conclusions:**

Adolescents with CKD are at risk for depression, anxiety, obsession, and panic disorders. Also, crying in tears/yelling response may be at greater risk for anxiety among CKD adolescents. Early psychiatric evaluation and routine psychiatric follow-ups initiated early may improve the mental health of this vulnerable population.

**Graphical Abstract:**

**Figure:**
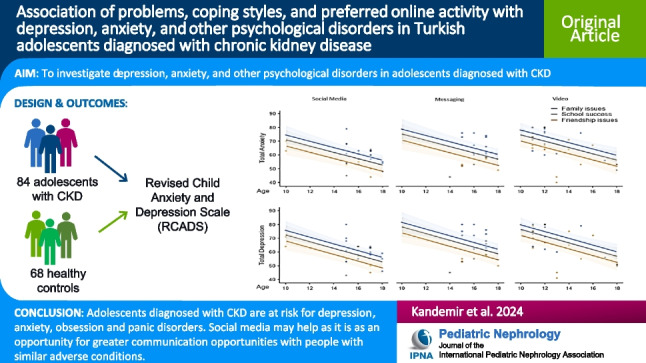
A higher resolution version of the Graphical abstract is available as Supplementary information

**Supplementary Information:**

The online version contains supplementary material available at 10.1007/s00467-024-06391-9.

## Introduction

Chronic kidney disease (CKD) refers to a progressive and irreversible loss of glomerular filtration due to structural kidney damage [1]. Beyond the challenges of the disease process and complications, psychiatric and psychosocial outcomes also come forth in children with CKD, and even their families present depressive symptoms [2]. Sudden onset illnesses and hospitalizations in childhood are risk factors that may harm mental health starting from childhood and extending to adulthood [3]. The depressive state is also a negative factor for poor neurocognitive performance in patients with CKD diagnosis [4]. Yet, depression and anxiety are related to worse life quality in adulthood [5], which might have its antecedents from childhood.

Children with chronic diseases should be treated in a multidisciplinary manner, especially with mental and social support, as their disadvantage may increase with psychological effects and school education interruptions [6]. Clinicians need to detect possible anxiety, depression, and other psychological disorders in them, so families can maintain acceptable life comfort.

A newly evolved reality of life, social media, is investigated by several studies and is reported to be a tool that may affect adolescents’ mental health unfavorably [7]. However, other studies suggest that behaviors on social media bring the potential to assess depressive mental health smoothly [8]. Despite that, some studies suggest that it may be helpful for adolescents by giving them the ability to make friends and reach easily accessed support [9]. Therefore, it is noteworthy to recognize the relationship between the psychological status scores of adolescents with CKD and their digital usage types.

A recent review emphasized that emotional, behavioral, social, and academic difficulties are common in children with CKD [2]. In the literature on this subject, especially adjustment disorders, depression, neurocognitive disorders, and anxiety disorders come to the fore [10]. However, the existence of psychological disorders such as social phobia, separation anxiety, and panic disorder has rarely been investigated. [11–13]. The Revised Child Anxiety and Depression Scale (RCADS) is a valid and confirmed cross-cultural assessment tool to assess anxiety, depression, and also other psychological disorders like obsessive behaviors, social phobia, separation anxiety, and panic disorder in childhood [14]. Its validity was confirmed by a study on Turkish children [15]. RCADS presents an opportunity for pediatricians to detect depression and anxiety disorders and send them to psychiatric specialists.

In this study, we assessed social phobia, separation anxiety, and panic disorder along with depression and anxiety in adolescents with CKD by RCADS and their associations with problems adolescents face, behavioral responses to problems, and preferred digital usage. In this way, we wanted to determine the risk of other psychological disorders that may be present in this patient group, the relationship between psychological status scores of adolescents with CKD and their digital usage preference, and to identify clues that would enable early detection of these disorders.

## Materials and methods

We conducted the study in the pediatric nephrology and adolescent medicine outpatient clinics of Istanbul University Faculty of Medicine in 2018. We included adolescents with stage 2 to 4 CKD who have a glomerular filtration rate that ranges from 15 to 89 ml/min/1.73 m^2^ and built an age- and gender-matched control group from healthy adolescents. We excluded the patients with a psychological disease history (patient or their family), under chronic treatment other than for CKD, have another chronic disease, failure to thrive, under psychiatric support, or have an acute illness that may impact the scores of the scales. We subjected all the participants to the RCADS scale, recording their age, gender, most problematic issues/topics in their lives (school success, family issues (divorce, disagreements, health problems, and financial problems), and friendship issues), their problem-coping strategies (crying in tears/yelling, enduring in silence, quarreling), and which applications they preferred most in their leisure time (social media, messaging, video). The participants read and completed the RCADS in a separate room outside the clinic.

The RCADS has six subscales, which are described as general anxiety (6 questions), separation anxiety (7 questions), panic disorder (9 questions), social phobia (9 questions), obsession (6 questions), and depression (10 questions). Each answer has four choices: never, sometimes, often, and always (counted as 0, 1, 2, and 3 points, respectively). A total score of 70 points and above scores was defined as elevated [16].

We estimated we would need a minimum of 64 patients in each group (two-tailed Student’s *t*-test, effect size d of 0.5, an error probability of 5%, and power (1-b error probability) 80%) to compare RCADS scores between the CKD and control groups. We included 70 patients to compensate for data loss and excluded two because of missing data. The other model was linear regression, which we planned to use to investigate the RCADS results with possible confounders. We estimated we would need 75 patients (*F* test, 80% probability of 1-b error, an error probability at 5% rate, effect size of *f*^2^ < 0.2, and with 6 predictors). We included 85 patients in each group in case of missing data, and we saw later that one patient in the CKD group did not complete the survey.

We obtained study permission from the Istanbul University Faculty of Medicine ethical committee with the file number 2018/202 and adhered to the Helsinki Declaration. All participants and their parents were informed, and their written consent was obtained to participate in this study.

### Statistical analysis

We used mean ± standard deviation and percentage results to present the data. We used the Shapiro–Wilk test to test normality and the Mann–Whitney *U* test to compare non-normally distributed groups (age and RCADS results between CKD and the control groups). To compare two categorical datasets, we used the chi-square test (for gender).

We built multivariate models (for RCADS results) and assessed significant confounders and used linear regression test (LR) if the assumptions (Durbin-Watson test for autocorrelation, Goldfeld-Quandt for heteroskedasticity, and variance inflating factor for collinearity) are met; otherwise, we used *generalized linear models* (GLM). We used the omnibus ANOVA test for LR and the loglikelihood ratio for GLM to calculate likelihood ratios. We assessed possible significant factors by the *backward elimination method* and set the significance threshold to *p* < 0.1 in the multivariate models for these subjected factors (age, gender, issues, problem coping type, and application preferences).

We would emphasize that the total expected results of subscales in GLM will increase with additional risk factors. For example, the mean expected panic disorder scale result is 66 in patients with CKD. However, this result will increase by 2.2 points among 13-year-old patients (compared with 15-year-old) and another 3.2 points in the female gender, resulting in a total of 71–72 points with a rough calculation.

We used the Jamovi 2.3.18 statistical package program with the “Gamlj” extension for calculations and graphics and performed minor revisions to the figures for better presentation. Also, we presented the strength of the multivariate models (with *R*^2^), confidence intervals (95%), and the estimated marginal means table. A type 1 error of *p* < 0.05 was considered for significance.

## Results

We included 84 adolescents with CKD (mean age 14.8 ± 2.4 years) and 68 healthy (mean age 14.3 ± 2.3 years) matched controls. There were no statistically significant differences between the two groups regarding gender (*p* = 0.253, chi-square test) and age (*p* = 0.153, Mann–Whitney *U* test).

### Comparing CKD and the control group

The comparison of the elevated results (defined as a score ≥ 70) is presented in Table 1. All the scales except general anxiety and social phobia were significantly elevated in patients with CKD compared to the control group, yet the general anxiety and total social phobia scores were statistically higher in the CKD group.

**Table 1:** Comparison of raw results and elevated scale results between CKD and the control group

	CKD (*n* = 84)		Control (*n* = 68)		*p*	
	Scores	Elevated	Scores	Elevated	Scores^*^	Elevated^**^
Separation anxiety	64 ± 11	29.8%	47 ± 6	0%	< 0.001	< 0.001
General anxiety	62 ± 6	3.6%	54 ± 4	0%	< 0.001	0.115
Panic disorder	66 ± 11	38.1%	53 ± 5	1.5%	< 0.001	< 0.001
Social phobia	51 ± 8	0%	40 ± 10	0%	< 0.001	N/A
Obsession	62 ± 13	34.5%	48 ± 11	4.4%	< 0.001	< 0.001
Depression	64 ± 12	36.9%	49 ± 8	1.5%	< 0.001	< 0.001
Total anxiety	63 ± 10	26.2%	48 ± 9	1.5%	< 0.001	< 0.001
Total depression	64 ± 11	34.5%	49 ± 9	1.5%	< 0.001	< 0.001

All the scores are given as mean ± standard deviation for presentation, and the ratio of elevated results as a percentage. The results were defined as elevated if the RCADS score ≥ 70

*N/A* not applicable as all the results were non-elevated

^*^Mann–Whitney *U* test

^**^Chi-square test

We built multivariate models with CKD and the control groups and subjected gender and age as covariates to assess confounders, and presented the estimated marginal means table with significant confounders in Table 2.

**Table 2:** Estimated marginal means tables of the scales regarding CKD, age, and gender

	Group		Age (year)			Gender		
	CKD	Control	13	15	17	Female	Male	*R*^2^
Separation anxiety	64 ^(63–66)^	47 ^(46–49)^	–	–	–	57 ^(55–59)^	55 ^(53–56)^	0.485
General anxiety	62 ^(60–63)^	54 ^(53–56)^	–	–	–	–	–	0.309
Panic disorder	66 ^(64–68)^	53 ^(52–55)^	62 ^(60–63)^	59 ^(58–61)^	57 ^(55–59)^	61 ^(59–63)^	58 ^(57–60)^	0.414
Social phobia	51 ^(50–53)^	40 ^(38–41)^	–	–	–	–	–	0.299
Obsession	62 ^(61–64)^	48 ^(46–49)^	57 ^(55–58)^	54 ^(53–56)^	52 ^(51–54)^	57 ^(55–59)^	53 ^(52–55)^	0.311
Depression	64 ^(62–65)^	49 ^(47–51)^	58 ^(56–59)^	56 ^(55–57)^	54 ^(52–56)^	–	–	0.367
Total anxiety	63 ^(61–65)^	48 ^(47–50)^	57 ^(56–59)^	55 ^(54–56)^	54 ^(52–55)^	57 ^(55–59)^	54 ^(53–56)^	0.414
Total depression	64 ^(63–66)^	49 ^(48–51)^	58 ^(57–60)^	56 ^(55–58)^	54 ^(53–56)^	58 ^(56–60)^	56 ^(54–57)^	0.410

Estimated means are estimated by keeping constant other effects in the model to the mean. The decimals are rounded up for presentation. 95%CI of all results are given in parentheses

**Fig. 1:**
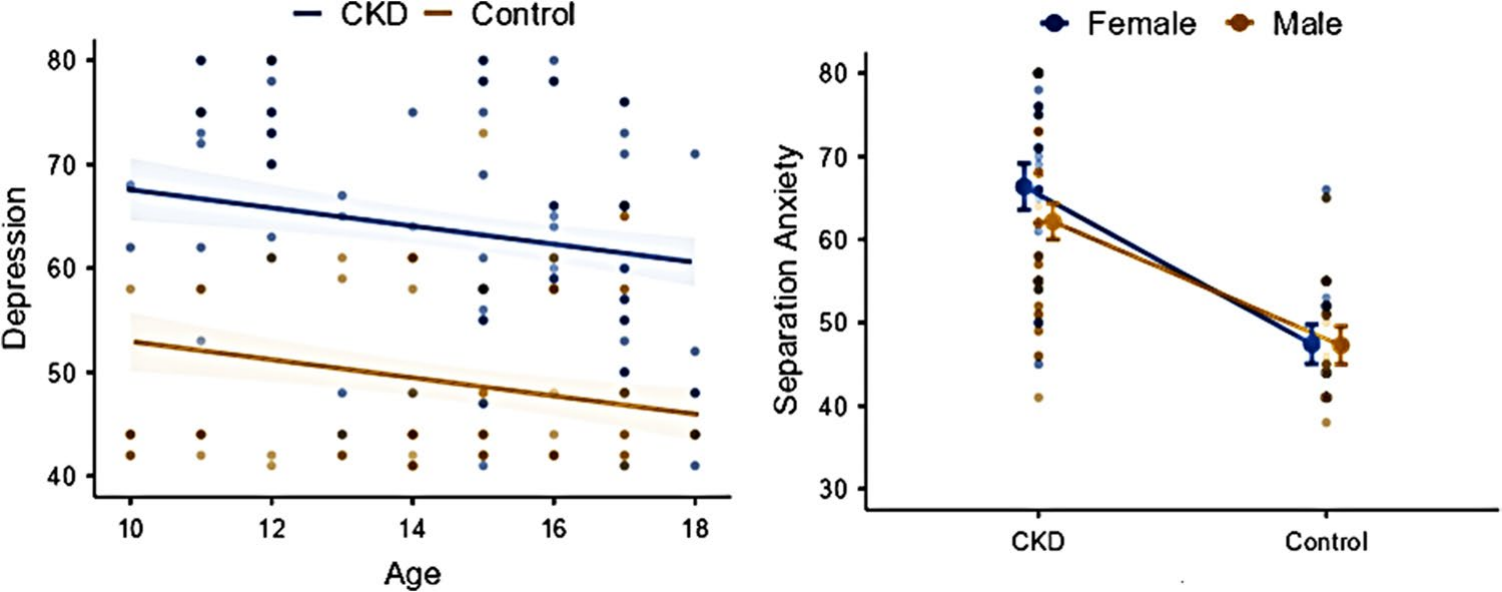
The association of chronic kidney disease (CKD), age, and gender with the depression and separation anxiety scales

We assessed significant confounders in RCADS scale results with the backward elimination method. The separation anxiety score increased by 16.9 points (95%CI 14.5–19.3) in the CKD group and 2.2 points (95%CI − 0.2 to 4.6) in girls compared with boys (GLM, R^2^:0.492) (Fig. 1, Table 2).

The general anxiety score was higher by 7.4 points (95%CI 5.0–9.9), and the social phobia score was higher by 11.9 points (95%CI 9.7–14) in the CKD group compared with the control group. The depression score was higher by 14.7 points (95%CI 12.3–17.1) in the CKD group and increased by 0.9 points (95%CI 0.4–1.4) per year decreasing in age. The total anxiety score was higher by 15.3 points (95%CI 12.9–17.7) in the CKD group and higher by 2.5 points in girls (95%CI 0.1–4.9), and increased by 0.7 points (95%CI 0.2–1.3) per year decreasing in age. The total depression score was higher by 15.5 points (95%CI 13.1–17.9) in the CKD group and by 2.4 points (95%CI 0–4.9) in girls and increased by 0.9 points (95%CI 0.3–1.4) per year decreasing in age (Fig. 1, Table 2). The panic disorder score was higher by 13.0 points (95%CI 10.5–15.5) in the CKD group and by 3.2 points (95%CI 0.8–5.7) in girls, and increased by 1.1 points (95%CI 0.5–1.6) per decreasing year in age. The obsession score was higher by 15.3 points (95%CI 12.9–17.7) in the CKD group and by 3.8 points (95%CI 1.5–6.2) in girls and increased by 0.9 points (95%CI 0.4–1.4) per decreasing year in age (Table 2). We should emphasize that estimated means were estimated by keeping constant other effects to the mean. Therefore, the expected RCADS score increases with the increasing number of risk factors.

### Assessing the utmost vulnerable sub-population among CKD patients with multivariate analysis

We built multivariate models and assessed if age, gender, social media preferences, consumed time, coping type with problems, problematic area, age, and gender were significant confounders.

**Fig. 2:**
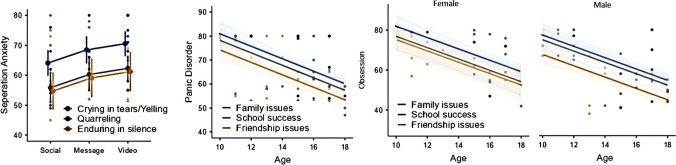
The association of age, gender, issues, and coping type with problems with the results of separation anxiety, obsession, and panic disorder scales

We assessed significant confounders with the backward elimination method in all scales and reported the remaining factors with *p* < 0.1 in likelihood ratio tests. Separation anxiety scale results were lower in social media preferring children by 4.4 points (95%CI − 1.0 to 9.7) and 6.5 points (95%CI 1.5–11.4) compared with messaging and video applications, respectively. Also, separation anxiety results were higher by 8.2 points (95%CI 3.6–12.8) and by 9.3 points (95%CI 3.2–15.5) in children with crying in tears/yelling response compared with children quarreling and enduring in silence, respectively (Table 3, Fig. 2). General anxiety scale results were higher in girls by 3.2 points (95%CI − 0.2 to 91.8) and increased by 0.9 points (95%CI 0.2–1.7) per year decrease in age. Panic disorder results increased by 2.6 points (95%CI 1.8–3.4) per year decrease in age, and was higher in children with family issues by 7.1 points (95%CI 3.1–11.0) and 2.9 points (95%CI − 1.9 to 7.6) compared with children with friendship issues and school success issues, respectively (Table 3, Fig. 2). Social phobia scale results were higher in crying in tears/yelling group by 4.2 points (95%CI 0.8–7.6) and 2.4 points (95%CI − 2.2 to 7.1) compared with quarreling and enduring in silence groups, respectively. In addition, per year decrease in age increased the social phobia scores by 1.1 points (95%CI 0.4–1.7). Obsession scale results were higher in girls by 4.8 points (95%CI 1.1–8.6), and increased by 2.9 points (95%CI 2.2–3.6) per year decrease in age. Also, family issues increased the obsession scale results by 3.4 points (95%CI − 1.4 to 8.1) and 9.4 points (95%CI 5.4–13.5) compared with school success issues and friendship issues, respectively (Fig. 2, Table 3).

**Table 3:** Estimated marginal means table of the scales regarding age, gender, problem coping type, most problematic issues, and application preference

	Age (year)			Gender		Problem coping type			Most problematic issue			Application preference			
	12	15	17	Girl	Boy	Cry	Qua	Sil	Fam	Sch	Frd	Soc	Mes	Vid	*R*^2^
Separation anxiety	–	–	–	–	–	68 ^(65–71)^	60 ^(56–63)^	58 ^(53–64)^	–	–	–	58 ^(55–62)^	63 ^(59–67)^	65 ^(61–68)^	0.227^*^
General anxiety	65 ^(62–67)^	62 ^(60–64)^	60 ^(58–62)^	64 ^(61–66)^	60 ^(58–63)^	–	–	–	–	–	–	–	–	–	0.197^**^
Panic disorder	73 ^(70–75)^	65 ^(63–67)^	60 ^(57–62)^	–	–	–	–	–	69 ^(66–72)^	66 ^(62–70)^	62 ^(59–65)^	–	–	–	0.304^**^
Social phobia	54 ^(51–57)^	51 ^(49–53)^	49 ^(46–51)^	–	–	53 ^(51–55)^	49 ^(46–52)^	51 ^(47–55)^	–	–	–	–	–	–	0.175^*^
Obsession	70 ^(67–73)^	61 ^(60–63)^	56 ^(53–58)^	65 ^(62–68)^	60 ^(58–62)^	–	–	–	66 ^(64–69)^	63 ^(59–67)^	57 ^(54–60)^	–	–	–	0.391^**^
Depression	70 ^(66–74)^	63 ^(61–65)^	58 ^(55–62)^	–	–	–	–	–	67 ^(63–70)^	64 ^(59–69)^	60 ^(56–64)^	59 ^(55–63)^	68 ^(64–72)^	64 ^(60–68)^	0.330^*^
Total anxiety	69 ^(65–72)^	62 ^(60–64)^	58 ^(55–61)^	65 ^(62–68)^	61 ^(59–63)^	–	–	–	66 ^(63–69)^	63 ^(59–67)^	59 ^(56–62)^	60 ^(57–63)^	64 ^(61–68)^	64 ^(61–67)^	0.407^**^
Total depression	70 ^(67–74)^	63 ^(61–65)^	58 ^(56–61)^	–	–	–	–	–	68 ^(65–70)^	64 ^(60–68)^	60 ^(57–62)^	60 ^(57–64)^	66 ^(63–70)^	64 ^(61–68)^	0.371^**^

Estimated means are estimated by keeping constant other effects in the model to the mean. The decimals are rounded up for presentation. 95%CI of all results are given in parentheses

*Cry.* crying in tears/yelling, *Qua.* quarreling, *Sil.* enduring in silence, *Fam.* family issues, *Sch.* school success, *Frd.* friendship issues, *Soc.* social media, *Mes.* messaging, *Vid.* video

^*^Linear regression test

^**^Generalized linear model

**Fig. 3:**
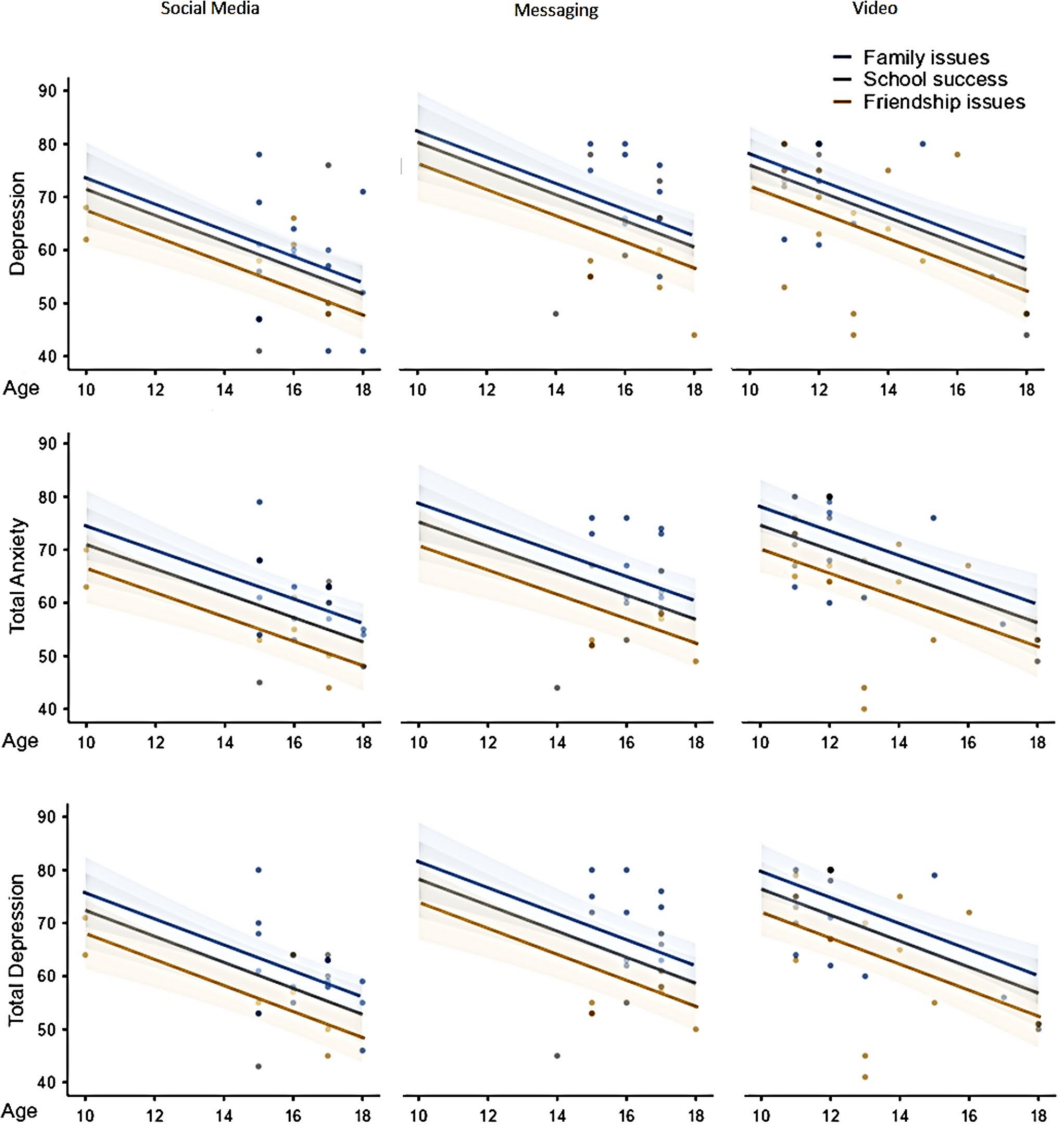
The association of problems and preferences with the results of general depression, total anxiety, and total depression results

Depression scale results were lower in social media preferring children by 9.1 points (95%CI 3.5–14.8) and 5.0 points (95%CI − 1.1 to 11.1) compared with messaging and video applications, respectively. Family issues increased depression scale results by 2.4 points (95%CI –3.5 to 8.4) and 6.7 points (95%CI 1.7–11.8) compared with school success and friendship issues, respectively. In addition, per year decrease in age increased depression results by 2.4 points (95%CI 1.2–3.6). Total anxiety results increased by 2.1 points per year decrease in age (95%CI 1.1–3.0) and was higher by 3.6 points (95%CI 1.1–3.0) in girls. Also, family issues increased depression scale results by 2.8 points (95%CI − 2.1 to 7.6) and 6.9 points (95%CI 2.8–11.1) compared with school success and friendship issues, respectively. In addition, depression scale results were lower in social media preferring children by 4.4 points (95%CI 0–8.8) and 4 points (95%CI − 0.8 to 8.8) compared with messaging and video applications, respectively. Total depression scale results increased by 2.5 points (95%CI 1.5–3.4) per year decrease in age, and family issues increased the results by 3.5 points (95%CI − 1.4 to 8.2) and 8.0 points (95%CI 4.0–12.0) compared with school success and friendship issues, respectively. Also, total depression scores were lower in social media preferring children by 5.9 points (95%CI 1.4–10.3) and 4.0 points (95%CI − 0.9 to 8.9) compared with messaging and video applications, respectively (Fig. 3, Table 3).

## Discussion

Anxiety and depression are not infrequent in children with chronic illnesses. Chronic kidney disease might be life-disturbing, with hospital visits, challenging therapy procedures, complications, and other unexpected circumstances. Therefore, not only anxiety and depression but other psychological problems should be investigated in this patient population. Also, social and digital media have both negative and positive impacts on adolescents. So, we aimed to determine which psychological problems affect adolescents with CKD and the ways they cope with these problems regarding behavioral or social/digital media platform preferences.

Anxiety is a common problem in adolescents with CKD. A study conducted with 8 to 12-year-old children under routine hemodialysis therapy reported higher anxiety than the control group [17]. Similarly, Elorza et al. [18] reported high anxiety levels in adolescents with CKD. By contrast, Stahl et al. [19] suggested that anxiety scores were lower in children with CKD than in healthy ones. However, a meta-analysis reported anxiety symptoms at 31% and 42% ratios for children with CKD and children on hemodialysis therapy, respectively [20]. In contrast, a study performed by the Child Depression Inventory evaluated separation anxiety in children with CKD and found higher scores than in healthy children, but general anxiety was not statistically different between the groups [13]. In our study, separation anxiety, general anxiety, and total anxiety scores were statistically higher in adolescents with CKD than in the control group; but similarly, the elevated general anxiety rates were not different between the two groups. Another aspect of our study was that we found general anxiety scores higher in girls and at younger ages. Gender is known as a prominent factor in anxiety disorders [21], and a study reported that anxiety is more frequent in girls [22], where our results were concordant with this finding in comparison of the CKD group with the control group (separation and total anxiety) and within the CKD group (general anxiety). According to these results, we think that separation and total anxiety should be evaluated in these patient groups, especially in girls.

Another possible mood disorder seen among patients with CKD is depression [4]. Among patients with CKD, about 30% with anxiety symptoms have depression, which might be a result of sharing the same symptomatology [21, 23]. Ten percent of all children in the world have a psychiatric disorder; however, most of these patients have no psychiatric support or treatment [24]. A literature review revealed a study which reported that depressive symptoms were higher in children with CKD than healthy controls [13]. Other studies suggested that depression was more frequent in the CKD group [18, 19]. Depression rates were too high, up to 65% and 67% (mild, moderate, and severe) in children who need routine hemodialysis and peritoneal dialysis groups, respectively [25]. However, a study reported lower depression rates of 17% in children with CKD and young adults and 5% had elevated depressive symptoms [4]. In our study group, the depression scales (depression and total depression) were elevated in about 35%, comparable with the literature. We picked up patients who were not under and had never received psychiatric support, which might be the cause of the high rates, but we think depression remains a prominent concern in clinical practice.

Our results suggest that age and gender also might affect depression rates. As we investigated age and depression association in the literature, a study reported that depressive symptoms were higher in adolescents [26]. The gender of the child is also an influential factor regarding depression, as girls are affected more than boys [21]. In our study, the total depression rate was higher in girls in the whole study group, but we did not find a significant effect of gender regarding depression scales (depression and total depression) within the patient group diagnosed with CKD. However, depression rates decreased with age in our study. According to these data, age seems to be an influential factor in depression, but girls do not seem to be an under-risk subgroup within the patient group diagnosed with CKD.

As we focus on social functioning and panic dysfunctions, Berney-Martinet et al. reported a 15% rate of social competence issues in patients with CKD, higher than in healthy peers [12]. We found that younger ages and emotionally reactive children (crying in tears/yelling) had increased social phobia scores among CKD patients in our study group; however, there was no difference as defined by elevated results between the groups. Another study investigated panic disorder and found it to not alter significantly from healthy children [13], similar to our results. However, panic disorder was more frequent in adolescents with CKD compared with the control group. Family issues and young age increased the vulnerability among the patients with CKD in our study. A study reported that panic disorder has an association with separation anxiety [27]. As our separation anxiety results were higher in the CKD group, we concluded that this might affect the high panic disorder rates. These results suggest that adolescents with CKD do not present social phobia problems but exhibit high panic disorder rates. The under-risk sub-group for panic disorder was a young age, but the attention-grabbing entity was that the adolescents with higher panic disorder results reported that they had family issues.

There are limited studies about obsession, another mood dysfunction, in children with CKD. Yousefichaijan et al. [28] reported that obsessive–compulsive disorder rates were significantly higher in the CKD group than in healthy controls. In our study, girls and younger adolescents had higher scores on the obsession scale, and family issues/problems increased this more. So, it may be concluded that family issues increase mental health problems in adolescents with CKD.

As we assessed the background of the reactions against issues, we found data that emotional reactions were coping mechanisms affected by anxiety or depression, where bad emotion regulation might occur because of elevated anxiety or depression symptoms in children with CKD [21, 29]. If the children think that a situation is not under control, this might result in increased stress, which causes anxiety and depression [21]. Among the patient groups with CKD in our study, crying-in-tears/yelling children had more separation anxiety scores. In this aspect, adolescents diagnosed with CKD with crying in tears/yelling response against a problem shall undergo psychiatric evaluation for anxiety.

Another aspect of the mental health of patients with CKD is the family role, as the support of parents has an influential role in mental health [29]. Panic disorder, obsession, depression, total anxiety, and total depression scores were higher in the children with CKD encountering family issues than friendship issues and educational success in our study group. Children focus on messaging to share their problems and seek support they cannot get from their families. We concluded that adolescents diagnosed with CKD with family issues/problems, especially those who need communication and aid from others that they cannot obtain from their families, have more risk for mental health issues and should undergo psychiatric evaluation.

Peer support is also an influential factor in the mental health of patients with CKD, and relationships with friends decrease in patients on routine hemodialysis, and focusing on self-esteem might increase life quality and decrease anxiety and depression [29, 30]. Social media preferring children had significantly lower anxiety and depression scores (separation anxiety, total anxiety, depression, and total depression) than those who preferred messaging and video applications. Although there are studies about social media causing depression, it may serve as an opportunity for adolescents with CKD to form friendships by creating enhanced opportunities for communication with people with similar adverse conditions [7, 9]. We think this condition affected decreased anxiety and depression scores in our study group.

The last aspect we should mention is that social media might serve as an opportunity for setting friendships. However, younger kids should be appropriately monitored by their parents, and social media use should be limited to a time that does not interfere with adolescents’ sleep or physical activity [31].

## Conclusion

Adolescents with CKD diagnosis are at risk, not only for depression and anxiety disorders but also for obsession and panic disorder. Therefore, early psychiatric evaluation and routine psychiatric follow-ups initiated early may improve the mental health of this vulnerable population. Family issues/problems may worsen mental health due to the increased anxiety and depression scores, as well as obsession. Messaging applications are associated with depression and anxiety scores in adolescents with CKD, especially if the adolescent encounters family issues/problems. Depression and anxiety scores decreased with age in our study group, which might be a cultural outcome. Adolescents with CKD diagnosis with crying in tears/yelling response against a problem may also need early psychiatric evaluation for anxiety.

### Limitations of the study

Though the RCADS score was abnormal, these children did not have an evaluation by a psychologist, so they did not have a confirmed diagnosis of anxiety or depression. We did not include family income or parental education status. Regarding social media preferences and issues faced by adolescents, we did not use a validated survey, and we defined family issues as divorce, disagreements, health problems, and financial problems. Also, we did not assess social media impact profoundly. We excluded patients with comorbid conditions and a psychiatry history and did not include the underlying primary kidney disease.

### Supplementary Information

Below is the link to the electronic supplementary material.

Graphical abstract (PPTX 206 KB)

## Data Availability

Data could be shared upon reasonable requests.
