# Supplementary material for: Association of problems, coping styles, and preferred online activity with depression, anxiety, and other psychological disorders in Turkish adolescents diagnosed with chronic kidney disease
Source: Pediatr Nephrol. 2024 May 21;39(9):2779–88. doi: 10.1007/s00467-024-06391-9 (PMC11272670; doi:10.1007/s00467-024-06391-9)
Supplement: Supplementary file 1 — (PPTX 206 KB) [file 467_2024_6391_MOESM1_ESM.pptx]

## Slide 1
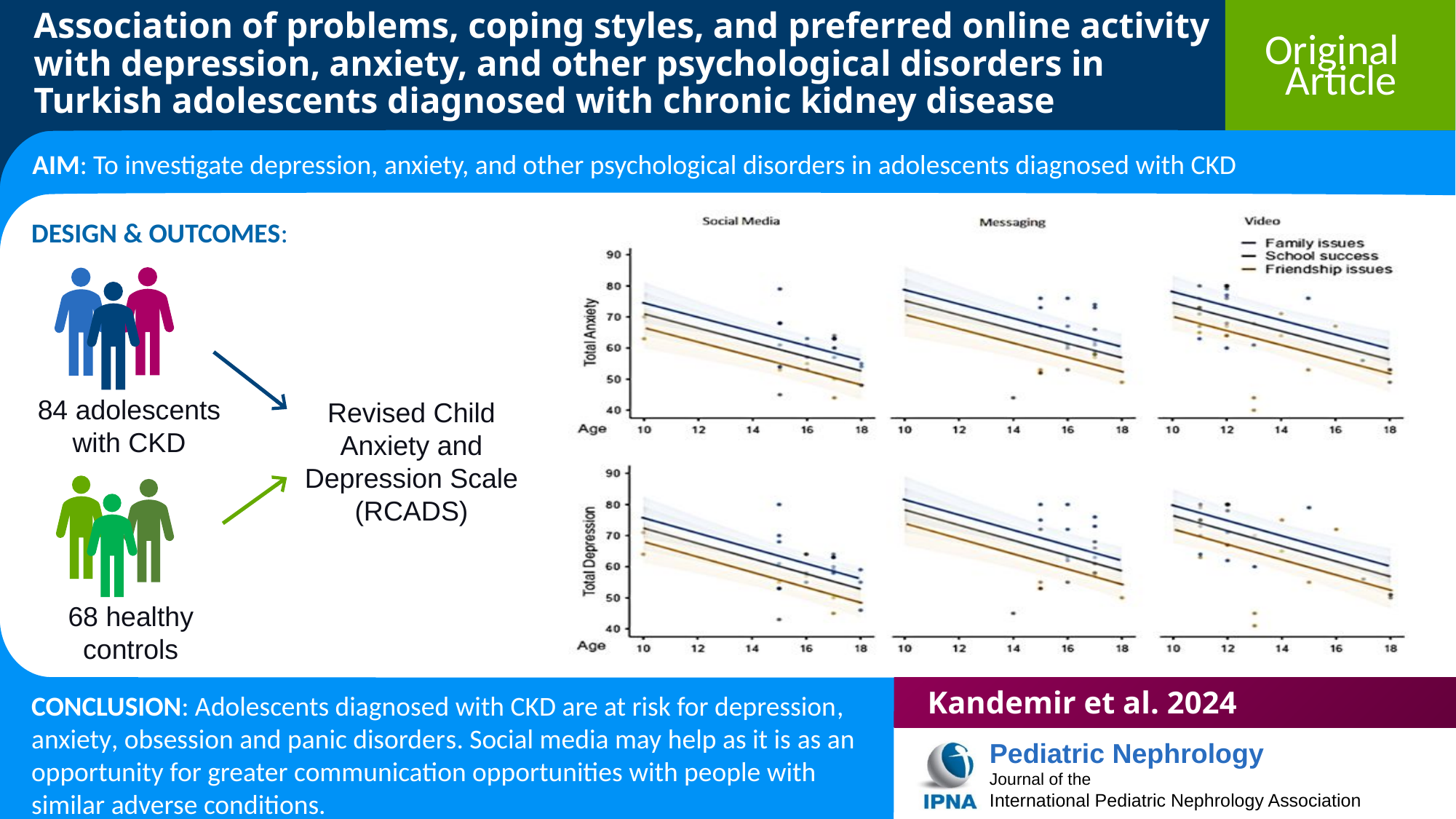

Association of problems, coping styles, and preferred online activity with depression, anxiety, and other psychological disorders in Turkish adolescents diagnosed with chronic kidney disease
AIM: To investigate depression, anxiety, and other psychological disorders in adolescents diagnosed with CKD
DESIGN & OUTCOMES:
84 adolescents with CKD
Revised Child Anxiety and Depression Scale (RCADS)
68 healthy controls
Kandemir et al. 2024
CONCLUSION: Adolescents diagnosed with CKD are at risk for depression, anxiety, obsession and panic disorders. Social media may help as it is as an opportunity for greater communication opportunities with people with similar adverse conditions.
